# CMR tagging for measurement of the long axis function and deformation rate of the systemic right ventricular free wall

**DOI:** 10.1186/1532-429X-11-S1-P101

**Published:** 2009-01-28

**Authors:** Sylvia SM Chen, Jenny Keegan, Andrew W Dowsey, Rick Wage, David N Firmin, Philip J Kilner

**Affiliations:** 1 Royal Brompton Hospital, London, UK; 2 Royal Brompton Hospital and Imperial College, London, UK; 3 The Brompton Hospital and Imperial College London, London, UK; 4 Imperial College, London, UK; 5 Imperial College London, London, UK; 6 The Brompton Hospital, London, UK

**Keywords:** Cardiac Magnetic Resonance, Right Ventricle, Deformation Rate, Great Artery, Right Ventricle Function

## Introduction

Although the systemic right ventricle (RV) in transposition of the great arteries generally functions well through childhood, it typically shows signs of failure later in life. Ejection fraction has been used as a cardiac magnetic resonance imaging (CMR) measurement of systemic RV function, but it is not easy to measure reproducibly due to complex geometry and prominent trabeculations, and may fail to detect early changes. Early detection of dysfunction could be advantageous, for example for trial and eventual implementation of a prospective pharmacological therapy.

## Purpose

We aimed to use CMR tagging of the free wall of the RV, with semi-automated analysis, as a measurement of RV long axis function, comparing findings with multislice CMR measurements of ejection fraction, in patients with Mustard operation for transposition of the great arteries (OTGA), or with unoperated congenitally corrected transposition of the great arteries (CCTGA).

## Methods

A breath hold four chamber steady state free precession cine dataset was acquired, followed by a breath hold cine gradient-echo echo-planar tagged acquisition in the same plane. Two pre-pulse labelled tag lines were applied immediately after the R wave, one across the basal RV myocardium and the other located 40 mm apical of the first. Motion of the tagged myocardium was tracked automatically by multi-resolution image registration so that the position of the tags, and their individual and relative displacements, could be derived throughout the cardiac cycle using semi-automated software. The RV free wall deformation rate was calculated by dividing the amount of deformation (the difference between the displacements of the basal and apical tag) by the time to maximum displacement of the basal tag (m/s).

## Results

Twelve patients (6 OTGA, age 30 ± 7 years and 6 CCTGA, age 35 ± 12 years) were studied and compared to 6 control subjects (age 31 ± 9 years). Basal RV myocardial displacements in both OTGA and CCTGA were reduced compared to control subjects (12 ± 5 and 20 ± 4 vs 25 ± 2 mm, p = 0.0003 and p = 0.03 respectively). Basal myocardial displacement in OTGA was more impaired than CCTGA (p = 0.007). The RV myocardial deformation rates in both OTGA and CCTGA were slower than in control subjects (0.03 ± 0.02 and 0.03 ± 0.02 vs 0.07 ± 0.02 m/s, p = 0.03 and p = 0.02 respectively). Ejection fractions did not differ significantly between patients and controls: OTGA 52 ± 7% and CCTGA 63 ± 12% vs control subjects 57 ± 4% (p = 0.2 and p = 0.5 respectively).

## Conclusion

RV free wall tagging by CMR with semi-automated analysis showed reduction of RV free wall motion and deformation rate in the patients relative to the controls in the absence of significant differences of volumetric ejection fraction. The reduction is likely to result from pressure loading in both patient groups. Greater reduction in OTGA than CCTGA may be a consequence of surgery in patients who had undergone atrial switch repair. RV free wall tagging could provide a relatively quick and simple method for longitudinal comparison of systemic RV myocardial function.

